# Non-invasive diagnosis of papillary thyroid microcarcinoma: a NMR-based metabolomics approach

**DOI:** 10.18632/oncotarget.13178

**Published:** 2016-11-07

**Authors:** Jinghui Lu, Sanyuan Hu, Paolo Miccoli, Qingdong Zeng, Shaozhuang Liu, Lin Ran, Chunxiao Hu

**Affiliations:** ^1^ Department of General Surgery, Qilu Hospital of Shandong University, Jinan 250012, P.R. China; ^2^ Department of General Surgery, University of Pisa, Pisa 56126, Italy; ^3^ Medical College of Shandong University, Jinan 250012, P.R. China

**Keywords:** papillary thyroid microcarcinoma, metabolomics, diagnosis, NMR

## Abstract

Papillary thyroid microcarcinoma (PTMC) is a subtype of papillary thyroid carcinoma (PTC). Because its diameter is less than 10 mm, diagnosing it accurately is difficult with traditional methods such as image examinations and FNA (Fine Needle Aspiration). Investigating the metabolic changes induced by PTMC may enhance the understanding of its pathogenesis and provide important information for a new diagnosis method and treatment plan. In this study, high resolution magic angle spin (HRMAS) spectroscopy and ^1^H-nuclear magnetic resonance (^1^H-NMR) spectroscopy were used to screen metabolic changes in thyroid tissues and plasma from PTMC patients respectively. The results revealed reduced levels of fatty acids and elevated levels of several amino acids (phenylalanine, tyrosine, lactate, serine, cystine, lysine, glutamine/glutamate, taurine, leucine, alanine, isoleucine and valine) in thyroid tissues, as well as reduced levels of amino acids such as valine, tyrosine, proline, lysine, leucine and elevated levels of glucose, mannose, pyruvate and 3-hydroxybutyrate in plasma, are involved in the metabolic alterations in PTMC. In addition, a receiver operating characteristic (ROC) curve model for PTMC prediction was able to classify cases with good sensitivity and specificity using 9 significant changed metabolites in plasma. This work illustrates that the NMR-based metabolomics approach is capable of providing more sensitive diagnostic results and more systematic therapeutic information for PTMC.

## INTRODUCTION

Thyroid nodules is a common clinical problem affecting 20-40% of the world population [1]. Most thyroid nodules are benign, and only 5%-10% are diagnosed as malignant [2]. A common endocrine tumor in head and neck area, thyroid cancer can be classified into four classes: papillary thyroid cancer (PTC), follicular thyroid cancer (FTC), medullary thyroid cancer (MTC), and anaplastic thyroid cancer (ATC) [3]. PTC, the most common and treatable class, accounts for 80% of the total thyroid cancer cases. For early stage PTC, 10-year survival rates can be as high as 90%, whereas for later stage PTC, 10-year survival rates are significantly lower [2-5]. Therefore, early diagnosis is crucial for better prognosis in PTC patients.

Fine Needle Aspiration (FNA) is the most common diagnostic method for thyroid cancer, and it can greatly assist doctors in distinguishing malignant tumors from benign ones. In most cases, FNA results are used to determine whether the patient will go through conservative treatment or surgical resection [5, 6]. FNA cannot distinguish follicular thyroid cancer from follicular adenomas, and 10-30 % of surgeries are determined to be unnecessary given the post-operational histological diagnosis [6-10]. FNA accuracy can also be affected by other factors, such as the size of the nodule and experience of the physician, which can significantly increase false negative rates, especially in patients with PTMC. Recent studies utilized several bio-molecular techniques to reduce false negative rates during the diagnosis. By incorporating genomic or proteomic markers such as BRAF [11-13], galectin-3 [14], E-cadherin, and CD44v6, accuracy can be effectively improved.

As part of a system biology approach, metabolomics is aimed at providing a comprehensive profile of all the metabolites present in a biological sample. Like other “Omics”, it has been applied in a broad range of applications, such as agriculture, environment monitoring, and medicine [15-17]. In recent years, metabolomics has been successfully applied in disease diagnosis [18]. In cancer research, Z. Huang *et al.* have used liquid chromatography-mass spectrometry based metabolomics in the early diagnosis of bladder and kidney cancer using urine as the sample [19]. Duartel *et al.* have utilized NMR based metabolomics techniques to discover biomarkers for lung cancer in urine [20].

However, little NMR based metabolomics research in thyroid cancer has been reported in last decade. Notably, Caldarelli's team used an HRMAS-NMR method to build a diagnostic model to discriminate malignant tumors from the benign ones [21, 22]. The resulting model has better sensitivity and specificity compared to the gold-standard FNA method. Another laboratory utilized ^1^H-NMR methods and focused on the metabolome of tumor tissue extracts [23]. Their model can also clearly distinguish normal tissue from benign nodules in FTC and PTC. These studies all focus on finding biomarkers in tumor tissues, and sample types such as plasma or urine has not yet been used for metabolomics research in PTMC.

The aim of the present study was to screen various metabolic changes and to discover significant changes in certain metabolites in thyroid tissue and plasma from PTMC patients by HRMAS and ^1^H NMR spectroscopy methods to create a diagnostic method and to predict clinical outcomes.

## RESULTS

### Histopathological evaluation of papillary microcarcinoma thyroid

**Figure 1:**
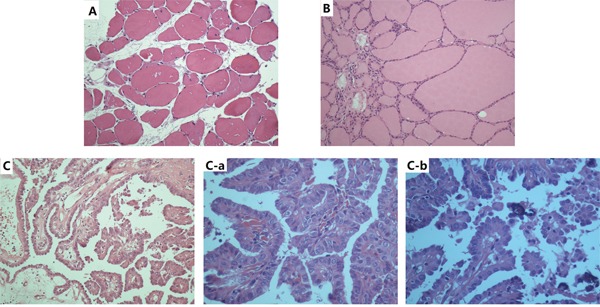
Representative HE-stained sections of thyroid ×200 **A.**, diffuse thyroid nontoxic goiter×200 **B.**, and papillary carcinoma×200 **C.**, ×400 (C-a), ×800 (C-b).

Other than papillary thyroid microcarcinoma, 2 cases of follicular carcinoma, 1 case of anaplastic carcinoma, and 6 cases of nodular goiter were also diagnosed in the 35 patients. Representative HE stained sections of thyroid from the patients are shown (Figure 1). Normal thyroid tissue showed clear lobules with follicles lined by flattened epithelium (Figure 1A). The nontoxic diffuse thyroid goiter showed colloid-rich follicles lined by flattened inactive epithelium, areas of follicular epithelial hypertrophy, and lymphocyte infiltration (Figure 1B). Papillary carcinoma showed a typically complex papillary architecture with branching, which are covered by epithelium with disturbed polarity and eosinophilic cytoplasm (Figure 1C). At the high power, the tumor presented typical overlapping, grooved (Figure 1Ca), ground glass nuclei with pseudoinclusion bodies and psammoma bodies (Figure 1Cb).

### HRMAS NMR based metabolomics of thyroid tissue between the PTMC group and the healthy group

**Figure 2:**
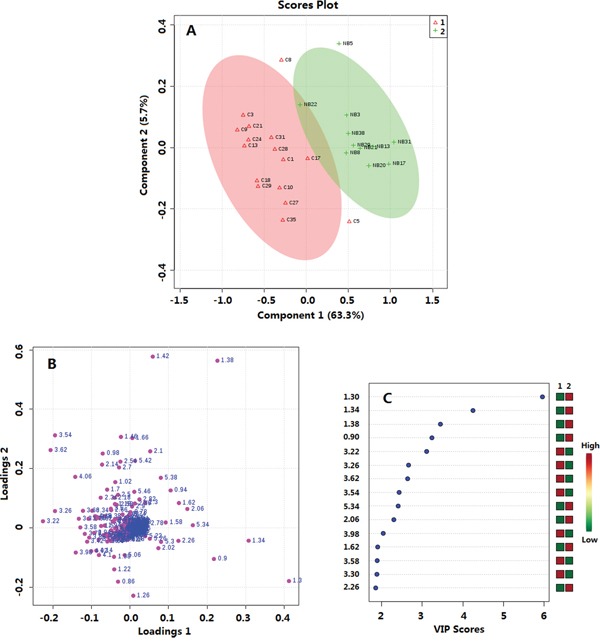
Multivariate data analysis of thyroid tissue metabolomics between PTMC and healthy groups **A.** OPLS-DA score plot, R2=0.84, Q2=0.76; **B.** Loadings plot; **C.** VIP scores. 1. PTMC groups; 2. Healthy groups

**Figure 3:**
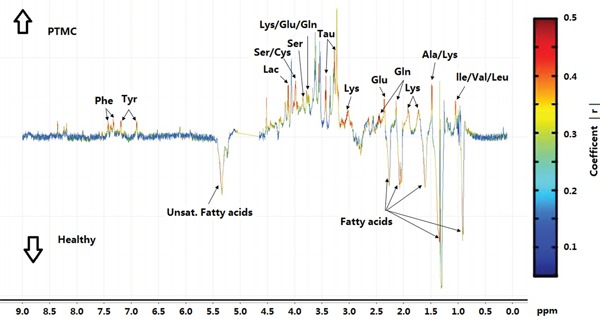
Coefficient-coded loading plots for the models discriminating between PTMC group and healthy groups Peaks in the positive direction indicate metabolites that are more abundant in the PTMC groups than healthy group (↑PTMC); Peaks in the negative indicate metabolites that are more abundant in the healthy group than PTMC group (↓Healthy).

By using the sectional integration method, the NMR spectral segments were all used for multivariable analysis. PLS-DA was used to explore the metabolic profiles of PTMC thyroid tissue and healthy thyroid tissue. Based on the ^1^H NMR spectra, clear discrimination was shown between them (Figure 2A). The parameters evaluating the PLS-DA model's validity, included an R2 of 0.84, a Q2 of 0.76 and *p* values <0.001, demonstrating that the PLS-DA models were robust and credible (Supplementary Figure S1). The PLS-DA loading plot suggested that the separation could be attributed to metabolites that have higher VIP value (VIP >1) and correlation value (|r| >0.4) (Figures 2, 3), including phenylalanine, tyrosine, serine, cystine, lysine, glutamine/glutamate, taurine, leucine, alanine, isoleucine, valine, fatty acids and lactate, compared with healthy group, saturated and unsaturated fatty acids with lower concentration and the others with higher concentration (Figure 3).

### ^1^H-NMR based metabolomics of plasma between PTMC group and healthy group

**Figure 4:**
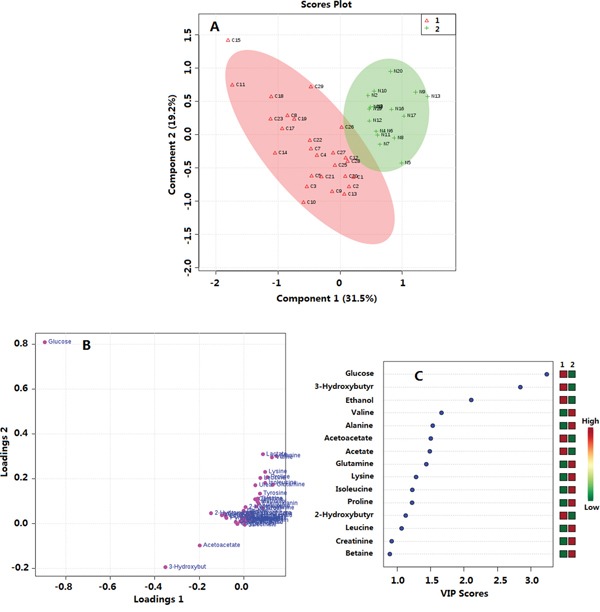
Multivariate data analysis of plasma metabolomics between PTMC and healthy groups **A.** PLS-DA score plot; R2 = 0.85, Q2 = 0.81 **B.** Loadings plot; **C.** VIP scores. 1. PTMC groups; 2. Healthy groups.

**Figure 5:**
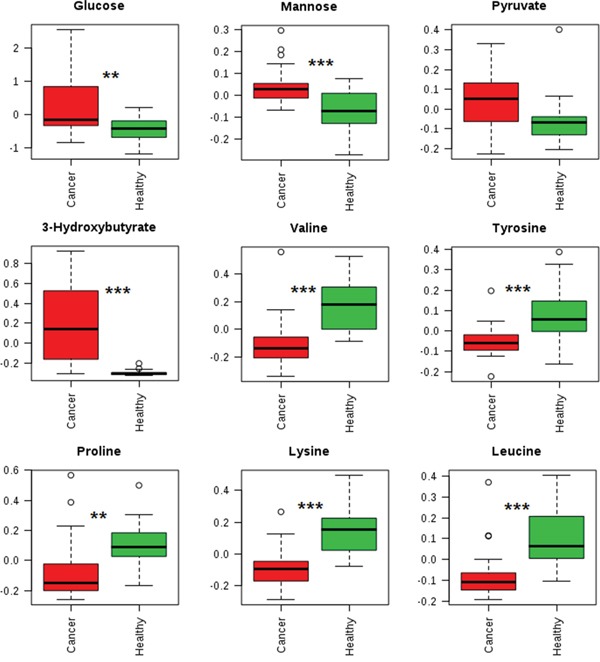
Box plots showing representative metabolite changes between PTMC and healthy groups **p < 0.01;***p < 0.001.

By using a targeted profiling method, 49 metabolites were identified and quantified (Supplementary Figure S2, Supplementary Table S1). All metabolites were used in the multivariable analysis. Clear discriminations of PTMC and healthy groups were observed in the PLS-DA score plots (Figure 4A). Two latent variables had performance values of R2 = 0.85, Q2 = 0.81. Additional permutation tests based on 1000 iterations were used to obtain *p* values of *p* <0.001 (Supplementary Figure S3). These validation plots assured the validity and robustness of the PLS-DA models. The PLS-DA loading plot visualized the distribution of 49 metabolites, and only those with a VIP of >1.0 was considered to be significant (Figure 4B, 4C). Accordingly, nine metabolites in plasma were considered, including glucose, mannose, 3-hydroxybutyrate, valine, tyrosine, proline, lysine, leucine, and pyruvate. Compared with the healthy group, the PTMC group had higher levels of glucose, mannose, and 3-hydroxybutyrate (*p* < 0.01; *p* < 0.001). Pyruvate level were higher in PTMC samples with no significant difference (*p* > 0.05), however, amino acids such as valine, tyrosine, proline, lysine, and leucine were all in significantly lower quantities in the PTMC samples (*p* < 0.01; *p* < 0.001) (Figure 5).

**Figure 6:**
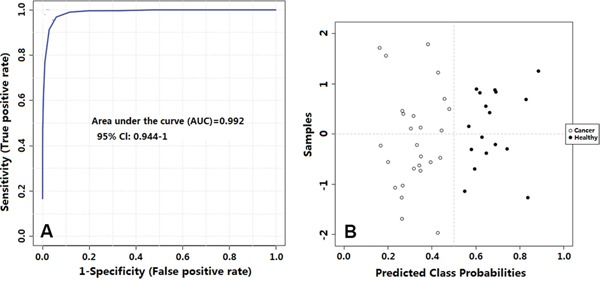
**A. Receiver operating characteristic curve showing PLS-DA model ability to predict thyroid tumor malignancy;B.** Predicted class plot showing the discrimination between thyroid lesions and their healthy counterpart tissues.

An ROC Curve analysis was used to evaluate the quality of this diagnostic model. A multivariable ROC curve was generated using the nine significant changed metabolites from plasma, which were glucose, mannose, 3-hydroxybutyrate, valine, tyrosine, proline, lysine, leucine, and pyruvate, and was built based on the PLS-DA model. The area under curve (AUC) was 0.992 with a Confidence Interval (CI) from 0.944 to 1 (Figure 6).

## DISCUSSION

In this metabolomics study of thyroid tissues, as compared with the healthy group, the PTMC group had lower levels of saturated and unsaturated fatty acids, and higher levels of phenylalanine, tyrosine, lactate, serine, cystine, lysine, glutamine/glutamate, taurine, leucine, alanine, isoleucine, and valine. These results were consistent with a previous report by Torregrossa et al [21]. Lower concentrations of fatty acids could be caused by an increase in metabolic rate and enhanced membrane synthesis in the cancer tissue [21]. Higher lactate levels in cancer tissue could be caused by activation of glycolysis.

In this metabolomics study of plasma, as compared with healthy group, the PTMC group had increased glucose, mannose, and pyruvate levels, and an increase in concentration of these compounds might be associated with upregulated glycolysis and enhanced amino acid catabolism [24]. As described above, amino acids undergo glucoeogenic and ketogenic pathways that could also contribute to blood glucose. The significant increase in 3-hydroxybutyrate, a precursor for fatty acid synthesis, in PTMC samples indicates that a higher rate of lipid synthesis might arise from altered lypolysis linked to the high-energy demands of these cells. These observations are also consistent with findings in other cancer research [24]. Amino acids such as valine, tyrosine, proline, lysine, and leucine were all significantly decreased in PTMC samples, which could have been due to increased protein synthesis in cancer patients. A decreased level of valine and leucine, which are branched-chain amino acids that can be converted into acetyl coenzyme A (Acyl-CoA) derivatives, in the plasma of PTMC patients indicated high rates of catabolism. The catabolism of valine and leucine leads to reduced coenzyme nicotinamide adenine dinucleotide levels and reduced flavin adenine dinucleotide levels, which can be utilized for adenosine triphosphate (ATP) generation, further suggesting that the decrease may be due to the strong demand of ATP by tumor cells. A reduction in proline and lysine levels is also observed, which might be a resulted by high rates of catabolism, and leading to an upregulated production of glutamate. Moreover, the concentration of tyrosine is found to significantly decreased in the plasma of PTMC samples, as tyrosine is equally important for protein biosynthesis as well as an intermediate in the biosynthesis of the catecholamines (dopamine, norepinephrine, and epinephrine). The changed metabolic status may be linked to altered metabolic pathways.

In addition, a ROC curve model for the prediction of PTMC using nine significant changed metabolites from plasma was able to classify cases with good sensitivity and specificity.

Taken together, metabolic reprogramming in PTMC is reflected by the highlighted metabolic markers. One of the emerging hallmarks that distinguish cancer from normal tissue is its metabolic reprogramming [24, 25]. The adjustments for energy metabolism and biosynthesis provide metabolites and cofactors required for cell growth and division [26]. The altered metabolic schemes clearly indicate an alternation between PTMC patients and healthy peoples not only at the tissue sample level, but also at the plasma sample level.

## CONCLUSION

In this study, we provided a novel metabolomics research on papillary thyroid microcarcinoma. Our findings highlight several metabolic markers associated with a significant change in the PTMC tissues.

Moreover, we demonstrated that plasma, a more systematic and accessible sample type, can also be used to construct a predictive model with high sensitivity and specificity for papillary thyroid microcarcinoma. This could be a great alternative tool for PTMC diagnosis. These results are promising as a method to improve the diagnosis, prognosis and management of patients.

The preliminary model is based on limited number of cases; therefore, larger cohorts should be studied to further validate these results. It would be insightful to validate connections between systematic and tissue specific metabolomics changes, and increase the number of sample cases in the future. It would be also interesting to examine other accessible samples, such as urine, and create a multivariable diagnosis model using metabolic biomarkers in this type of sample.

## MATERIALS AND METHODS

### Human patients selection and specimen collection

**Figure 7:**
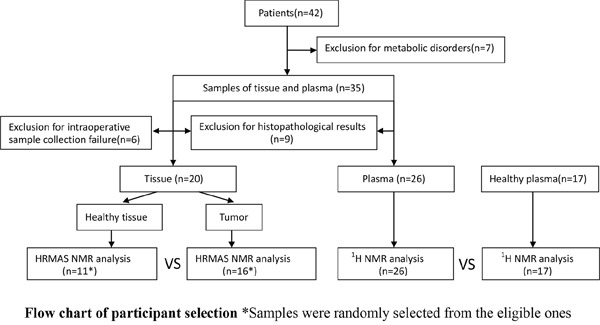
Flow chart of participant selection

This prospective study was approved by the institutional ethical review committee of Qilu Hospital, Shandong Province, China. All the specimens were obtained in Qilu Hospital. All participants were provided written informed consent for the use of their thyroid tissue and blood samples for research purposes. In December 2014, 42 consecutive patients (36 females and 6 males, 45.1± 8.5 yr) underwent total thyroidectomy or lobectomy in the department of general surgery for a single thyroid nodule less than 10 mm, and 20 volunteers (17 females and 3 males, 30±3.4 yr) were involved in this study. Thyroid nodules in 35 patients were preoperatively diagnosed as or suspected to be malignant by FNA according to the Bethesda classification. The FNA failed or was rejected in the remaining 7 patients, so the preoperative diagnosis was made based on image analysis. Participants who suffered from diabetes, hyperlipemia, hypertension, anaphylactic diseases or other metabolic diseases, or had a history of previous thyroid surgery or hormone treatment were excluded from the study, which included 7 patients and 3 volunteers. (Figure 7)

Samples of nodule tissue and nearby tissue (control specimens) were collected successfully from 29 patients during the surgical procedures, whereas in the remaining 6 patients, the size of the nodule was too small to ensure that sufficient tissue could be obtained after sample collection for subsequent histopathological examination. The tissue samples were frozen in liquid nitrogen immediately after harvest and stored at -80°C until HRMAS-NMR analysis.

Plasma samples were collected preoperatively from 35 patients and 17 volunteers prior to the morning meal with over 8 hours fasting. No intravenous or intramuscular injections were performed in the past three days. Samples were collected by a registered medical technician using a plasma-collecting tube with heparin as the anticoagulant in Qilu Hospital of Shandong University. Samples were then centrifuged at 3000 rpm for 5 min. Plasma was collected in a sterilized 1.5 mL centrifuge tube. The plasma samples were flash frozen in liquid nitrogen and stored at -80°C until NMR analysis.

### Histopathological examination

The formalin-fixed thyroid tissues were embedded in paraffin and then cut into 4-μ m sections. Thyroid sections were stained using hematoxylin and eosin (HE) or periodic acid-Schiff, and the slides were evaluated under light microscopy in whole thyroid sections by three pathologists specializing in thyroid disorders (mean:6.1 years, 4~15 years of experience).

### ^1^H-HRMAS NMR experiments of tissue samples

20 mg frozen thyroid tissue was weighed, placed into a centrifuge tube, and then quickly rinsed in 0.9% NaCl solution (99.9% deuterated water). Samples were placed in a 4 mm ZrO_2_ HRMAS rotor with 5 uL D_2_O added to it. All HRMAS experiments were carried out in a Bruker 800 MHz Avance III NMR spectrometer equipped with a 4mm ^1^H/13C/31P HR-MAS probe. Samples were spun at a rate of 8 kHz at 283 °K. ^1^H-HRMAS spectra were acquired by using a Carr Purcell Meiboom Gill (CPMG) NMR spin echo sequence to suppress the effect of macromolecules and lipids followed by a water pre-saturation pulse during relaxation time. For each sample, 64 transients were acquired with a spectral width of 12000 Hz and 32000 data points.

### ^1^H-NMR spectroscopy of plasma samples

Plasma was taken out from the -80°C freezer and thawed at 4°C. Then, 500 μL of plasma as added to a 3 KDa ultra filtration filter (Millipore, USA). The filter unit was centrifuged at 13000 rpm for 30 min at 4°C. Then, 450 μL filtrate was collected and mixed with 50 μL Anachro DSS Standard Solution (4.0260M DSS quantified in 99.6% D2O, Anachro Technologies Inc., Calgary, Canada). Solutions were vortexed for 30 s followed by 2 min centrifugation at 13000 rpm. 480 μL of the prepared sample was transferred into a 5 mm NMR tube (Norell Inc., Marion, USA). All plasma samples were run on a Bruker Avance III 600 NMR spectrometer equipped with Cryo Probe™. A MetNOESY pulse sequence was applied with 100 ms mixing time and 4 seconds acquisition time. 990 ms saturation pulse was used to suppress water signal during acquisition. 64 transients were collected for each sample.

### Spectrogram processing and multivariate pattern recognition analysis

For NMR spectra recorded in tissue samples, Spectra were phased, baseline corrected, and referenced to Alanine's signal at 1.47 ppm in Chenomx NMR Suite 8.1 (Chenomx Inc., Edmonton, Canada). Spectrum binning was performed on all ^1^H HRMAS data with a bin width of 0.04 ppm from 0.1 ppm to 10.00 ppm. In order to avoid interference caused by water suppression, the region from 4.65 ppm to 5.05 ppm was excluded. The resulting data matrix was normalized to the total area under spectrum curve. Metabolite signal assignment was performed in Chenomx NMR Suite 8.1 using a targeted profiling method with an internal database. Some ambiguous assignments were confirmed with ^1^H -^1^H COSY spectra, ^1^H -^1^H TOCSY spectra, and ^1^H-^13^C HSQC spectra. For NMR spectra recorded in plasma samples, spectra were phased, baseline corrected, and reference deconvoluted against the DSS singlet at 0 ppm using Chenomx NMR Suite 8.1. The targeted profiling method [27] was used to qualify and quantify all metabolite compounds in these spectra.

The normalized integral values from tissue samples and compound concentration data from plasma samples were then subjected to multivariate pattern recognition analysis using “PCA Methods” [28], “PLS” [29] package, and data visualization was performed using the “ggplot2” package [30] in the R programing environment. Principle Component Analysis (PCA) was first used to detect grouping trends and outliers. Partial least squares discriminant analysis (PLS-DA) was then performed for class discrimination and biomarker selection. Evaluation of the PLS-DA models was performed using the goodness of-fit parameter R2 (variation in class membership explained by the model) and the predictive ability parameter Q2 (goodness of prediction, calculated by 7-fold internal cross-validation), where values of R2 and Q2 close to 1.0 represent excellent modelling. In addition, a permutation test on the response (1000 random permutations) was also computed (P<0.001 means there is no random model found with better model quality, compared with the original one). Potential biomarkers were discovered according to variable importance in the project (VIP) value and the loading plot was generated from PLS-DA analysis. A Receiver Operating Characteristic (ROC) Curve was used to build a diagnosis model by an online metabolomics analysis platform, “MetaboAnalyst 3.0” [25] with the plasma metabolomics data.

### Statistical analysis

A Welch's t test was performed using “t.test” in R programing environment to determine the significance of each metabolite. *p*< 0.05 was considered to be statistically significant.

## SUPPLEMENTARY MATERIALS




