# Supplementary material for: Non-invasive diagnosis of papillary thyroid microcarcinoma: a NMR-based metabolomics approach
Source: Oncotarget. 2016 Nov 7;7(49):81768–77. doi: 10.18632/oncotarget.13178 (PMC5348428; doi:10.18632/oncotarget.13178)
Supplement: Supplementary file 1 [file oncotarget-07-81768-s001.pdf]

Non-invasive diagnosis of papillary thyroid microcarcinoma: a NMR-based metabolomics approach

SUPPLEMENTARY MATERIALS

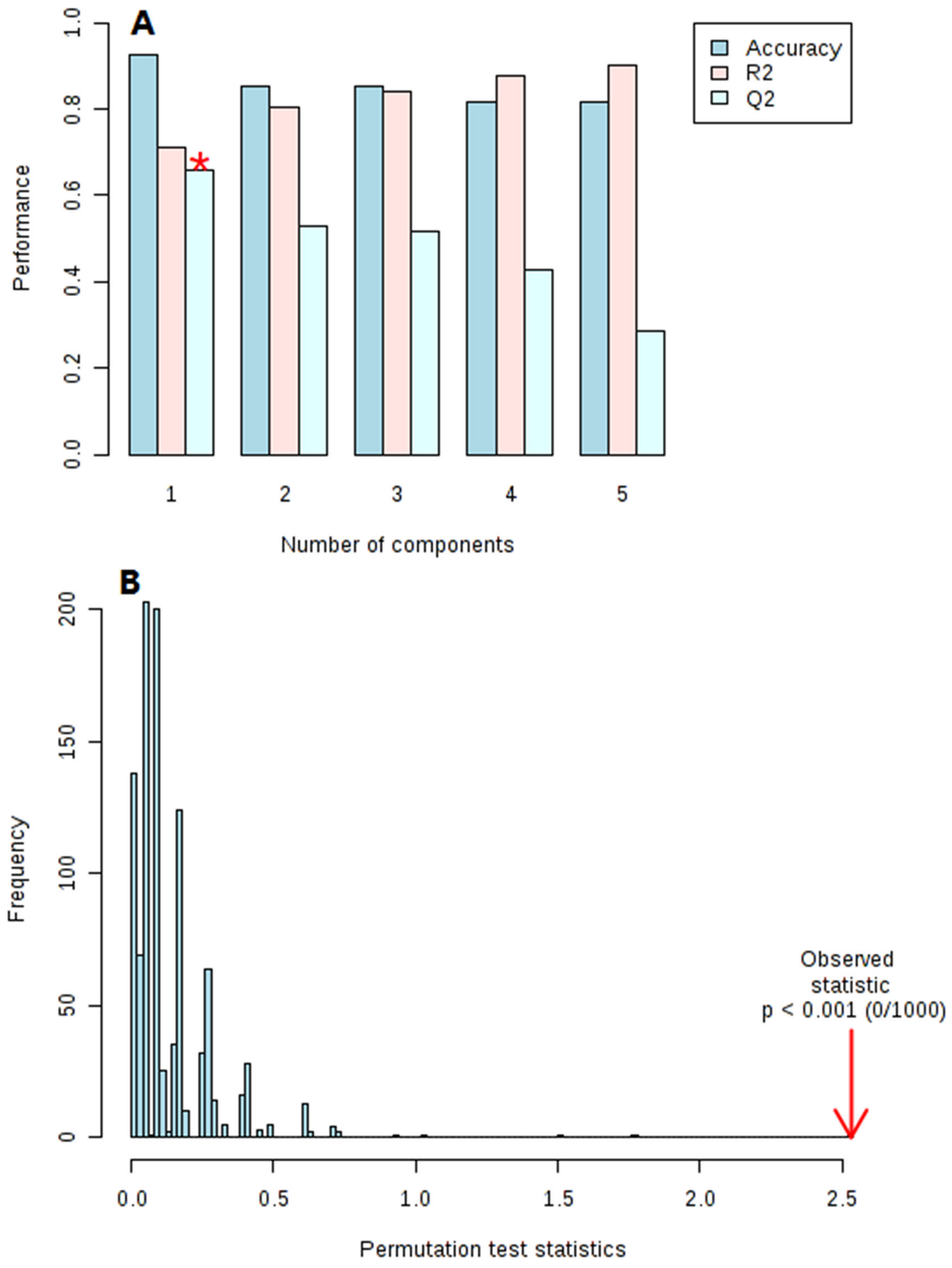

Supplementary Figure S1: A. PLS-DA classification using different number of components. The red circle indicates the best classier, R2=0.84, Q2=0.76; **B.** Permutation tests based on separation distance. The *p* value based on permutation is $p < 0.001$ (0/1000).

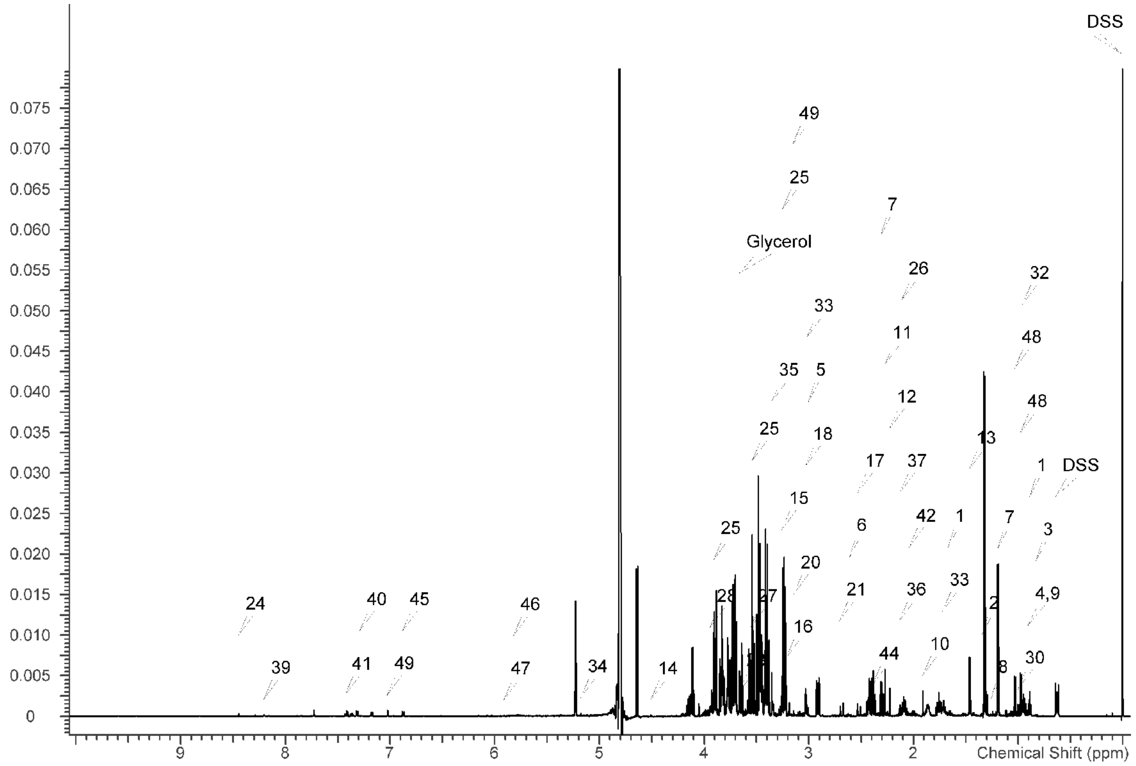

Supplementary Figure S2: Representative ^{1}H-NMR spectra of plasma samples. 1. 2-Hydroxybutyrate; 2. 2-Hydroxyisobutyrate; 3. 2-Hydroxyisovalerate; 4. 2-Hydroxyvalerate; 5. 2-Oxoglutarate; 6. 2-Oxoisocaproate; 7. 3-Hydroxybutyrate; 8. 3-Hydroxyisovalerate; 9. 3-Methyl-2-oxovalerate; 10. Acetate; 11. Acetoacetate; 12. Acetone; 13. Alanine; 14. Ascorbate; 15. Betaine; 16. Carnitine; 17. Citrate; 18. Creatine; 19. Creatinine; 20. Dimethyl sulfone; 21. Dimethylamine; 22. Ethanol; 23. Ethylene glycol; 24. Formate; 25. Glucose; 26. Glutamine; 27. Glycine; 28. Glycolate; 29. Isobutyrate; 30. Isoleucine; 31. Lactate; 32. Leucine; 33. Lysine; 34. Mannose; 35. Methanol; 36. Methionine; 37. O-Acetylcarnitine; 38. O-Phosphocholine; 39. Oxypurinol; 40. Phenylacetate; 41. Phenylalanine; 42. Proline; 43. Pyruvate; 44. Succinate; 45. Tyrosine; 46. Urea; 47. Uridine; 48. Valine; 49.τ-Methylhistidine.

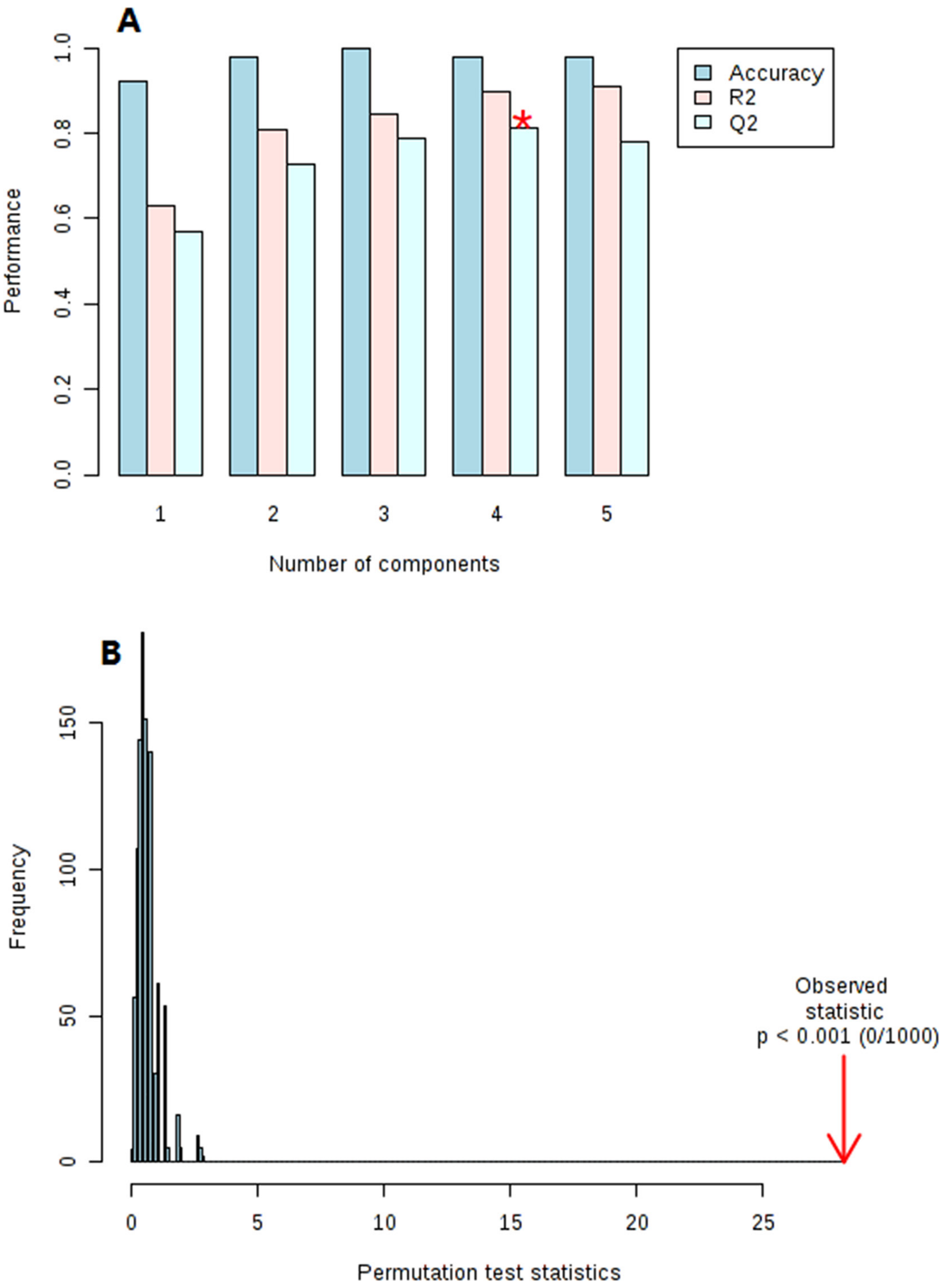

Supplementary Figure S3: A. PLS-DA classification using different number of components. The red circle indicates the best classier, R2 = 0.85, Q2 = 0.81; **B.** Permutation tests based on separation distance. The *p* value based on permutation is $p < 0.001$ (0/1000).

Supplementary Table S1: The concentration of 49 metabolites from plasma between PTMC and healthy groups

See Supplementary File 1
