# Supplementary material for: Non-invasive diagnosis of papillary thyroid microcarcinoma: a NMR-based metabolomics approach
Source: Oncotarget. 2016 Nov 7;7(49):81768–77. doi: 10.18632/oncotarget.13178 (PMC5348428; doi:10.18632/oncotarget.13178)
Supplement: Supplementary file 2 [file oncotarget-07-81768-s002.docx]

| Concentration | PMC | | Healthy | |
| --- | --- | --- | --- | --- |
|  | AVG | SD | AVG | SD |
| 2-Hydroxybutyrate | 0.059804 | 0.026743 | 0.028535 | 0.009564 |
| 2-Hydroxyisobutyrate | 0.001862 | 0.000562 | 0.001718 | 0.000528 |
| 2-Hydroxyisovalerate | 0.005969 | 0.002262 | 0.008029 | 0.004214 |
| 2-Hydroxyvalerate | 0.031427 | 0.014756 | 0.033147 | 0.00872 |
| 2-Oxoglutarate | 0.006058 | 0.002946 | 0.0091 | 0.003981 |
| 2-Oxoisocaproate | 0.004115 | 0.00146 | 0.004124 | 0.001328 |
| 3-Hydroxybutyrate | 0.213208 | 0.151489 | 0.023465 | 0.011993 |
| 3-Hydroxyisovalerate | 0.001223 | 0.000444 | 0.004112 | 0.001978 |
| 3-Methyl-2-oxovalerate | 0.003431 | 0.001051 | 0.003676 | 0.00109 |
| Acetate | 0.137712 | 0.175094 | 0.043547 | 0.00886 |
| Acetoacetate | 0.075962 | 0.052514 | 0.018841 | 0.006811 |
| Acetone | 0.028846 | 0.021332 | 0.017012 | 0.005805 |
| Alanine | 0.244558 | 0.063517 | 0.3169 | 0.058627 |
| Ascorbate | 0.009527 | 0.005031 | 0.011282 | 0.004866 |
| Betaine | 0.031881 | 0.010514 | 0.047641 | 0.009076 |
| Carnitine | 0.022896 | 0.010818 | 0.034753 | 0.009685 |
| Citrate | 0.072715 | 0.01577 | 0.061912 | 0.008717 |
| Creatine | 0.025604 | 0.009183 | 0.033488 | 0.020926 |
| Creatinine | 0.045823 | 0.007804 | 0.0632 | 0.009901 |
| Dimethyl sulfone | 0.004631 | 0.002119 | 0.004665 | 0.002214 |
| Dimethylamine | 0.000942 | 0.000147 | 0.001406 | 0.000385 |
| Ethanol | 0.297146 | 0.800944 | 0.011788 | 0.00398 |
| Ethylene glycol | 0.004612 | 0.001555 | 0.006441 | 0.001018 |
| Formate | 0.019454 | 0.005295 | 0.019241 | 0.002355 |
| Glucose | 3.893096 | 0.767686 | 3.261447 | 0.299248 |
| Glutamine | 0.260123 | 0.043822 | 0.321912 | 0.046868 |
| Glycine | 0.141673 | 0.049985 | 0.137418 | 0.037273 |
| Glycolate | 0.01255 | 0.004679 | 0.0115 | 0.004237 |
| Isobutyrate | 0.005638 | 0.002314 | 0.007629 | 0.002646 |
| Isoleucine | 0.041723 | 0.013752 | 0.072994 | 0.021287 |
| Lactate | 1.512838 | 0.641258 | 1.530947 | 0.404913 |
| Leucine | 0.0573 | 0.018988 | 0.087276 | 0.024025 |
| Lysine | 0.091596 | 0.019055 | 0.130035 | 0.02451 |
| Mannose | 0.031865 | 0.008496 | 0.0211 | 0.009233 |
| Methanol | 0.099592 | 0.027409 | 0.113106 | 0.025966 |
| Methionine | 0.014762 | 0.002602 | 0.021729 | 0.004922 |
| O-Acetylcarnitine | 0.006792 | 0.00317 | 0.004629 | 0.001594 |
| O-Phosphocholine | 0.005146 | 0.001946 | 0.0069 | 0.001713 |
| Oxypurinol | 0.053335 | 0.046403 | 0.043206 | 0.045611 |
| Phenylacetate | 0.001881 | 0.000453 | 0.002341 | 0.000828 |
| Phenylalanine | 0.034465 | 0.00598 | 0.0471 | 0.009725 |
| Proline | 0.112454 | 0.041814 | 0.152859 | 0.033087 |
| Pyruvate | 0.043223 | 0.020105 | 0.031171 | 0.020407 |
| Succinate | 0.006065 | 0.002219 | 0.006418 | 0.001342 |
| Tyrosine | 0.038888 | 0.009388 | 0.054765 | 0.017536 |
| Urea | 0.075527 | 0.029529 | 0.106841 | 0.038261 |
| Uridine | 0.003362 | 0.001242 | 0.002665 | 0.001136 |
| Valine | 0.157777 | 0.040472 | 0.225259 | 0.040892 |
| tao-Methylhistidine | 0.027592 | 0.010741 | 0.028924 | 0.010623 |
